# Beneficial effects of melittin on ovalbumin-induced atopic dermatitis in mouse

**DOI:** 10.1038/s41598-017-17873-2

**Published:** 2017-12-15

**Authors:** Woon-Hae Kim, Hyun-Jin An, Jung-Yeon Kim, Mi-Gyeong Gwon, Hyemin Gu, Minji Jeon, Woo Jung Sung, Sang Mi Han, Sok Cheon Pak, Min-Kyung Kim, Kwan-Kyu Park

**Affiliations:** 1 Department of Pathology, College of Medicine, Catholic University of Daegu, Daegu, Korea; 2 Department of Agricultural Biology, National Academy of Agricultural Science, Jeonju-si, Korea; 3 School of Biomedical Sciences, Charles Sturt University, Bathurst, Australia; 4 Department of Pathology, College of Medicine, Dongguk University, Gyeongju-si, Korea

## Abstract

Atopic dermatitis (AD) is an inflammatory skin disease characterized by intense pruritus and relapsable eczematous lesions. The hallmarks of AD are defects in the epidermal barrier and immunoglobulin E (IgE)-mediated sensitization to several environmental allergens, as well as an immune disorder mediated by an imbalance toward T-helper-2 response. Melittin, a major component of bee venom, has been studied in various inflammatory diseases. However, the beneficial effects of melittin on mouse with AD-like symptoms have not been explored. Therefore, we investigated the anti-allergic effects of melittin. AD was induced by ovalbumin (OVA) patch. After agent treatment, skin tissues and sera were extracted from the sacrificed mice were used to demonstrate the effects of melittin through various molecular biological methods. The results showed that OVA-induced skin thickening and inflammatory infiltration were decreased in the melittin-treated group. Melittin prevented OVA-induced filaggrin deficiency and imbalanced inflammatory mediators. Furthermore, melittin inhibited IL-4/IL-13-induced filaggrin downregulation through the blockade of STAT3 activation in human keratinocytes. In summary, this study has shown that melittin ameliorated OVA-induced AD-like symptoms from various perspectives. The findings of this study may be the first evidence of the anti-inflammatory effects of melittin on OVA-induced AD.

## Introduction

Atopic dermatitis (AD) is an inflammatory skin disease characterized by intense pruritus and relapsable eczematous lesions. It increases the risk for food allergy, asthma, allergic rhinitis, other immune-mediated diseases, and mental disorders^1^. The hallmarks of AD are defects in the epidermal barrier and immunoglobulin E (IgE)-mediated sensitization to several environmental allergens, as well as an immune disorder mediated by an imbalance toward T-helper-2 (Th2) response^2^. The mechanism involved in AD has not been clearly understood, although it has been studied for a long time. Two hypotheses have been proposed. One is that the primary disturbance resides in an immunologic disorder that induces IgE-mediated sensitization, with epidermal barrier dysfunction considered a result of local inflammation, and the other is that an intrinsic defect in the epithelial cells causes epidermal barrier dysfunction; the immunologic aspects are considered a consequence of the barrier dysfunction^3^. The primary events and key drivers of AD are topics of continuing argument^4^. However, both hypotheses point out defects in the epidermal barrier and the immune system; the skin barrier function and the immune system closely interact. Therefore, an effective AD therapy may need to target not only epidermal barrier but also immune function.

The skin is an efficient physical, chemical, microbial, and immunological barrier that performs various protective functions in the epidermis. The stratum corneum is a major epidermal barrier structure. Several abnormalities in the stratum corneum are well-established features of AD, such as increased water loss and decreased hydration^5–7^. Filaggrin is one of the main structural components of the stratum corneum, and it contributes to the keratin cytoskeleton as a template for the assembly of the cornified envelope. From a genetic standpoint, null mutation in filaggrin is the strongest known genetic risk factor for AD^8^. Except for inherited filaggrin null mutations, various factors, such as filaggrin copy number variants, mechanical damage, low humidity, and a cutaneous cytokine imbalance in AD, contribute to a decrease in filaggrin expression^9^. Filaggrin deficiency affects several pathways that are relevant for epidermal barrier dysfunction^10,11^, and it is also associated with subclinical inflammation^12^, increased permeability to low molecular weight, and water-soluble tracers^13^. Thus, an increase in filaggrin expression may have a crucial role in the amelioration of the stratum corneum, and it may contribute to the recovery of the skin barrier function and to cutaneous inflammation.

Cutaneous inflammation in AD is characterized by gradual and sequential patterns of inflammatory cell infiltration, especially by the cluster of differentiation (CD)4^+^ cells^14^. Infiltrating T cells cause the expression of several skin-homing adhesion molecules, such as chemokines, lymphocyte antigen, and lipid chemoattractant receptors, which can induce them to recruit into cutaneous areas^15^. Furthermore, increased Th2 cytokine-producing type-2 innate lymphocytes are detected in AD lesions and contribute to local inflammation^16^. The progression of AD is characterized by a thickened epidermis with sprouting of nerve fibers, increased expression of immunostimulatory chemokines, and obvious infiltration by Th2 cells and dendritic cells^17,18^. Thymic stromal lymphopoietin (TSLP) is important inflammatory mediator in AD. TSLP is derived from fibroblasts and keratinocytes, contribute to tissue renovation and fibrosis, and it also induces the activation of dendritic cells, drives Th2-polarization, and enhances B-cell differentiation and proliferation^19^. Cutaneous inflammation and epidermal barrier dysfunction are closely related and mutually reinforced^1^. Disruptions in the epidermal barrier activate keratinocytes to produce chemokines that attract T cells, cytokines that mediate innate immune reactions (such as interleukin (IL)-1 family), and cytokines that trigger Th2 polarization and Langerhans cell activation (such as TSLP)^20^. Much evidence shows that the interaction of the disrupted epidermal barrier with compromised immune responses also supports percutaneous allergic symptoms^21,22^.

Pruritus is the most important AD symptom^3^. The disordered interaction of immune cells and keratinocytes is also important in pruritus. Upon the activation of these cells, both produce an amount of mediator that causes sprouting of nerve fibers, and stimulate sensory nerve endings^23^. To promote itch, TSLP derived from keratinocytes communicates directly with cutaneous sensory neurons^24^, and TSLP contributes Th2 cells to produce cytokines, such as IL-13, which stimulate nerve fibers^25^. Therefore, a decrease in the expressions of TSLP and IL-13 is crucial to ameliorate pruritus in AD.

In AD, inflammatory epidermal dendritic cells and Langerhans cells, which have a receptor for IgE, lead to antigen presentation^26^. Through IgE bound to the receptor, these cells can take up allergens that cause IgE-mediated responses of the prompt type, and enhance T-cell-mediated responses of the delayed type^26^. Antigen-specific IgE plays an important role as the recognition structure for allergens on mast cells^3^. IgE-mediated sensitization causes degranulation of mast cells, and the released granules contribute to allergenic symptoms^27^. In the AD animal models we used, repeated epicutaneous sensitization with ovalbumin (OVA) induces OVA-specific IgE and eczematous lesions similar to those in AD^28,29^.

No known complete cure for AD exists at present; only multistep approaches that improve symptoms and achieve long-term disease control are available^1^. One of the main principles in AD therapy is anti-inflammatory therapy with topical corticosteroids, representing the first-line anti-inflammatory and most potent treatment for AD^1^. Phototherapies and immunosuppressants, such as calcineurin inhibitor cyclosporine A, are also useful treatments for AD^30,31^. These therapies have been well established to reduce inflammation, but they can cause side effects^31^. Developing new therapeutic approaches, which have a low risk of side effects, for AD is therefore necessary.

Melittin is the main component (50% of dry weight) of bee venom^32^. Bee venom is a natural toxin produced by honeybee (*Apis mellifera*), and it has been widely used as a traditional therapy in Oriental clinics^33^. Nowadays, several pieces of evidence have demonstrated that bee venom has pharmacological activities for various diseases^34^. Melittin is a cationic, hemolytic peptide, and it has anti-bacterial, anti-viral, and anti-inflammatory activities^35^. In previous studies, melittin induced cell cycle arrest and apoptosis in various cancer cells^36,37^, and showed beneficial effects on mouse models of liver cirrhosis^38^, atherosclerosis^39^, and *Propionibacterium acnes*-induced inflammation^40^.

Although these studies on skin disease showed that melittin has useful effects, they were inadequate in demonstrating the beneficial effects of melittin on AD *in vivo*. Therefore, the present study examined the beneficial effects of melittin as an alternative therapy for AD *in vivo*. Melittin ameliorated OVA-induced AD-like symptoms in mice, such as increased skin thickness with inflammatory infiltrate, reduction of filaggrin, production of AD-related inflammatory cytokines and chemokines, and exaggerated IgE response. These results indicate the therapeutic effects of melittin on AD-like symptoms.

## Results

### Melittin prevents OVA-induced AD-like skin lesions

**Figure 1:**
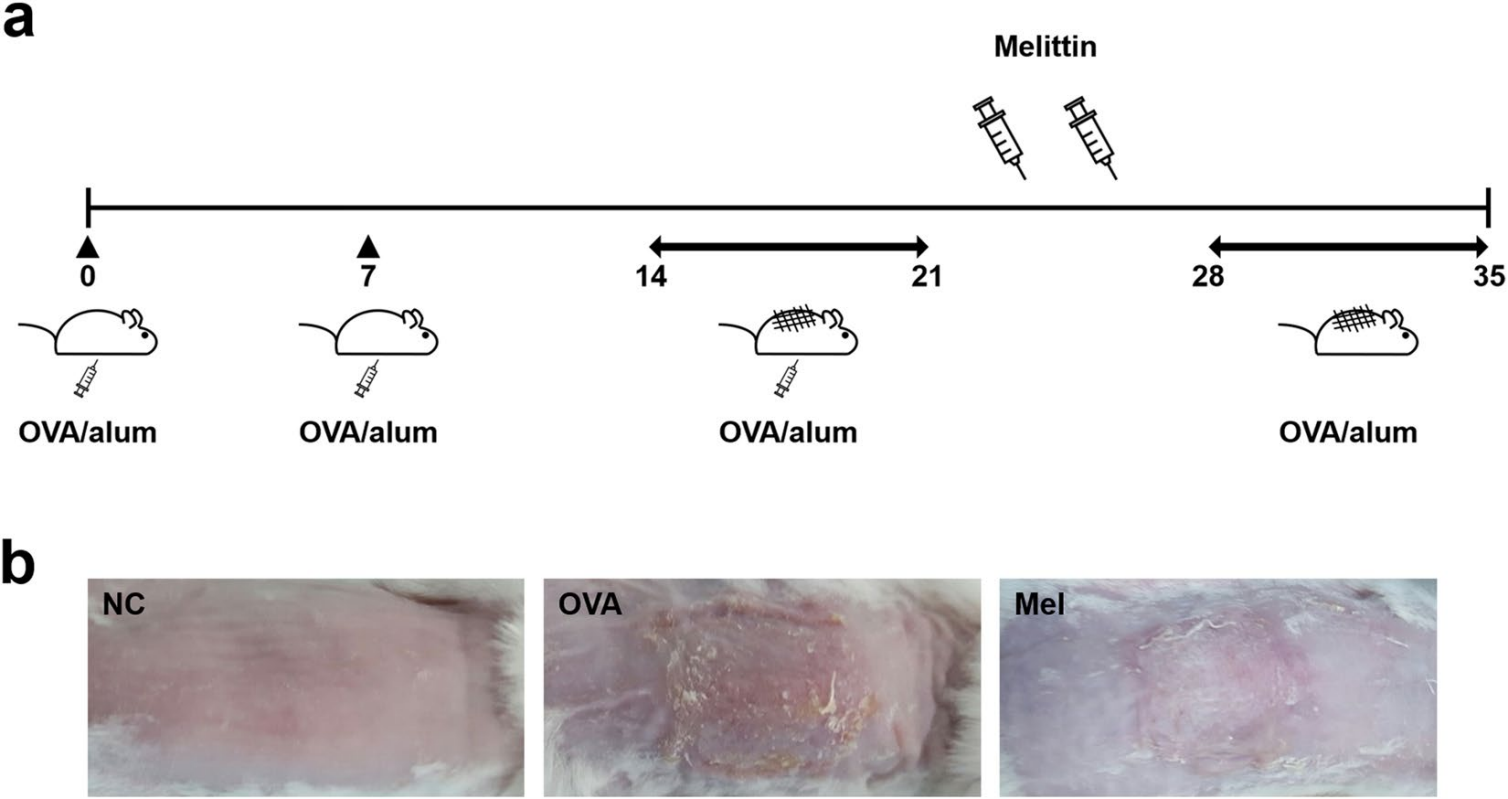
Scheme of AD induction and melittin treatment. (**a**) The mice in the group without NC were intraperitoneally inoculated with OVA/alum on days 0, 7, and 14, and epicutaneously stimulated with OVA patch on days 14 and 28 for 1 week each. Melittin was injected intraperitoneally between the epicutaneous OVA stimulation procedures. (**b**) Mouse dorsal skin lesions of each group. NC: normal control; OVA/alum: ovalbumin and aluminum hydroxide. Mel: 100 μg kg^−1^ melittin.

**Figure 2:**
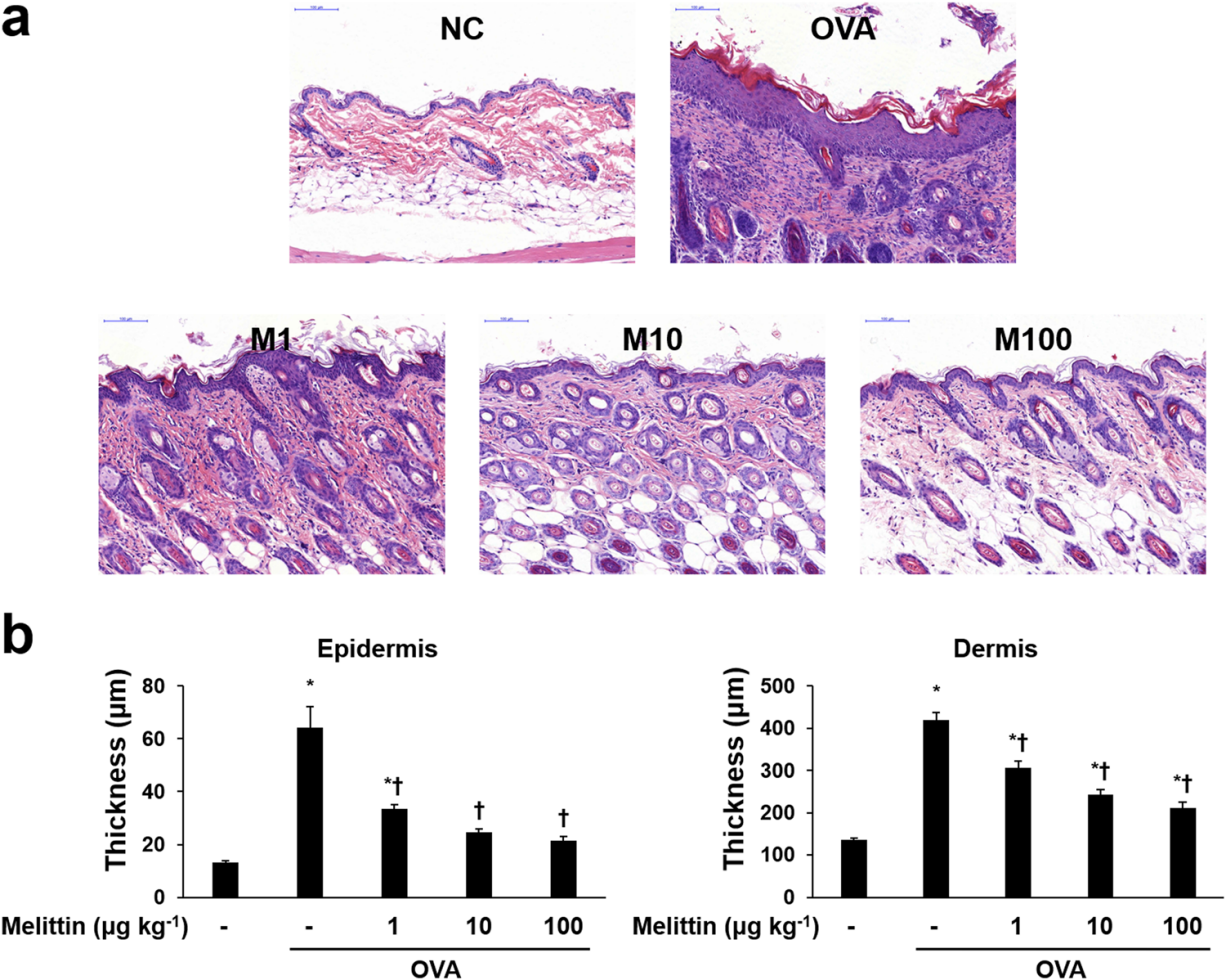
Effects of melittin on OVA-induced inflammatory infiltrate and skin thickening. (**a**) Representative images of histologic analysis with H&E staining show that melittin ameliorated the OVA-induced inflammatory infiltrate and the thickening of the epidermis and dermis. Scale bar = 100 μm. (**b**) The thicknesses of the epidermis and dermis were measured from at least 10 random fields per section at 200-fold magnification. Results are expressed as means ± SEM. *p < 0.05 compared with the NC group. ^†^ *p* < 0.05 compared with the OVA group. NC: normal control; OVA: ovalbumin; M1, M10, and M100: 1, 10, and 100 μg kg^−1^ melittin; −: untreated.

After AD induction, the skin conditions of the mice seemed to have worsened such as edema, erythema, and excoriation in the OVA group, but they were improved in the melittin group. (Fig. 1b). Hematoxylin and eosin (H&E) staining was performed to analyze the inflammatory infiltrate and the thicknesses of the epidermis and dermis, and at least 10 random fields per slide were selected. Each thickness was measured linearly and perpendicular to each surface of skin specimen. The OVA stimulation increased the inflammatory infiltrate and made the mouse skin thickened. The skin thickness in the melittin groups was thinner than that in the OVA group (Fig. 2).

### Melittin ameliorates OVA-induced filaggrin deficiency

**Figure 3:**
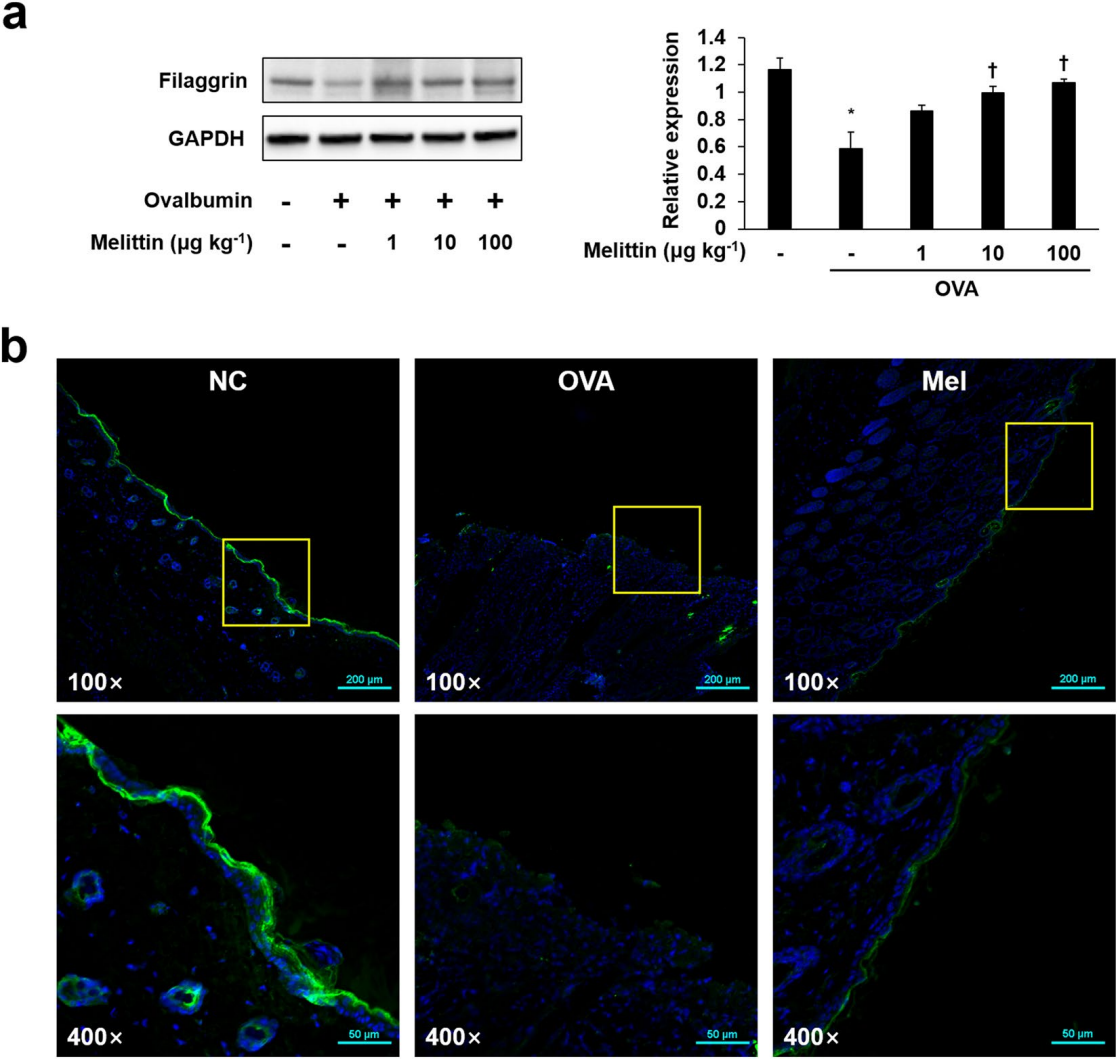
Melittin improved OVA-induced filaggrin deficiency. (**a**) Western blot analysis shows filaggrin and GAPDH protein expressions in the mouse dorsal skins of each group. GAPDH was used to confirm equal sample loading. The bar graph shows the quantitative signal intensity of filaggrin after normalization with GAPDH. The images are representative of three independent experiments. The results are expressed as means ± SEM. *p < 0.05 compared with the NC group. ^†^ *p* < 0.05 compared with the OVA group. +: treated; −: untreated. The samples were derived from the same experiment and that blots were processed in parallel. The blots were cropped and full-length the blots can be found in Supplementary Fig. S1a (**b**) Representative immunofluorescence images show the expressions of filaggrin (labeled with Alexa Fluor 488, green). The nuclei were labeled with Hoechst 33342 (blue). NC: normal control; OVA: ovalbumin; Mel; 100 μg kg^−1^ melittin; 100×: 100-fold magnification; 400×: 400-fold magnification. The scale bars of the three upper images are 200 μm, whereas those of the three bottom images are 50 μm.

Filaggrin deficiency plays a crucial role in barrier dysfunction, as mentioned in the introduction part. Filaggrin deficiency was confirmed by performing Western blot and immunofluorescence analysis. The expression of filaggrin was decreased in the OVA group compared with the normal control (NC) group. However, the expression of filaggrin was enhanced in the melittin groups in a dose-dependent manner, especially for the 100 μg kg^−1^ melittin group that showed the highest filaggrin expression value (Fig. 3a). We also performed immunofluorescence analysis to determine whether 100 μg kg^−1^ melittin ameliorates filaggrin deficiency in paraffin-embedded skin specimens. In the NC group, filaggrin expression was high and connected linearly, whereas it was almost absent in the OVA group. The expression of filaggrin was higher in the melittin group than in the OVA group (Fig. 3b).

### Melittin inhibits OVA-induced CD4 and CD11b expressions in the mouse dorsal skin

**Figure 4:**
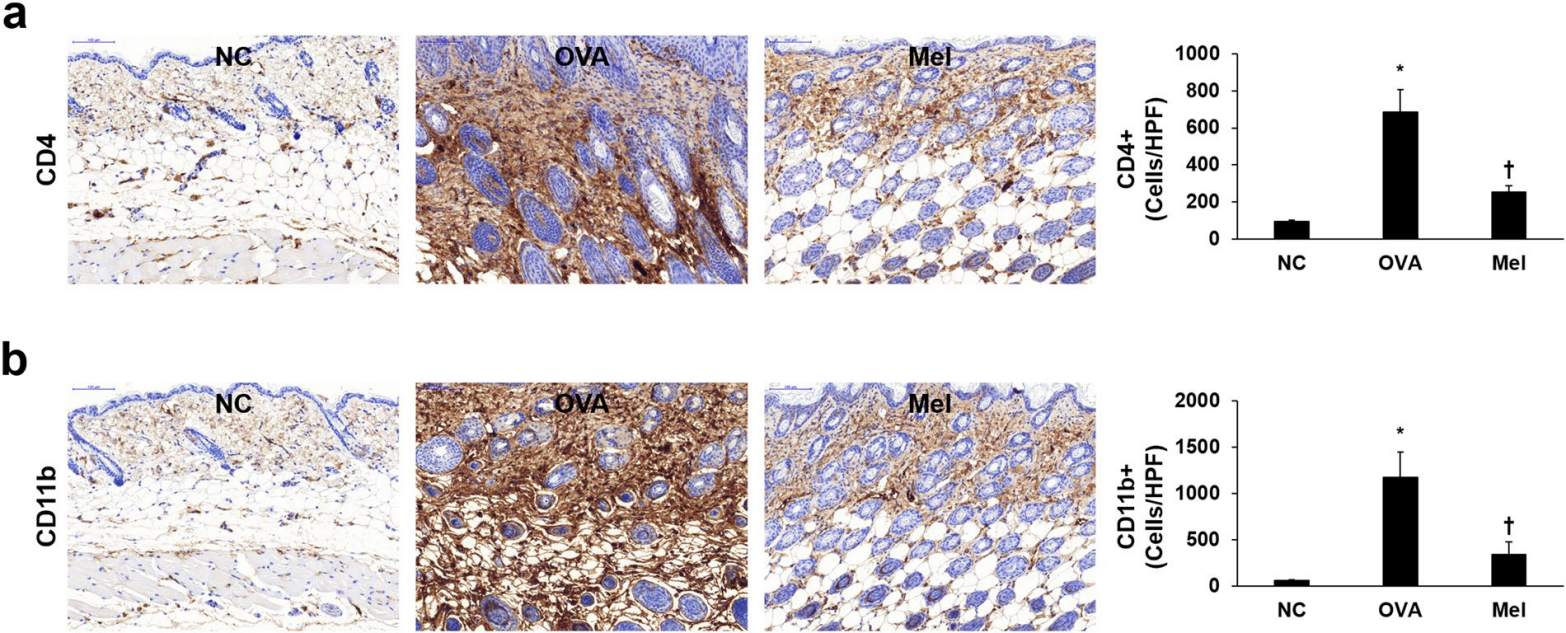
Distribution of CD4+ and CD11b+ cells in mouse dorsal skin sections. Representative immunohistochemical analysis with CD4 and CD11b antibodies shows the expressions of CD4 (**a**) and CD11b (**b**), indicated in brown color. The integrated optical densities were measured from at least five random fields per section at 200-fold magnification, respectively. Results are expressed as means ± SEM. *p < 0.05 compared with the NC group. ^†^ *p* < 0.05 compared with the OVA group. NC: normal control; OVA: ovalbumin; Mel: 100 μg kg^−1^ melittin; HPF: high-power field.

Infiltration of immune cells, such as CD4+ T helper cells, monocytes, macrophages, and dendritic cells, is an important histopathological change in AD. CD11b, also known as macrophage-1 antigen, is expressed on the surface of many leukocytes, including monocytes, granulocytes, and macrophages^41^. We confirmed the OVA-induced infiltration of immune cells and the effect of melittin by immunohistochemical staining of CD4 and CD11b. The expressions of CD4 and CD11b in dorsal skin were significantly increased in the OVA group compared with the NC group. However, the OVA-induced expressions of CD4 and CD11b were significantly decreased in the 100 μg kg^−1^ melittin group compared with the OVA group (Fig. 4).

### Melittin decreases OVA-induced mast cell infiltration and IgE expression

**Figure 5:**
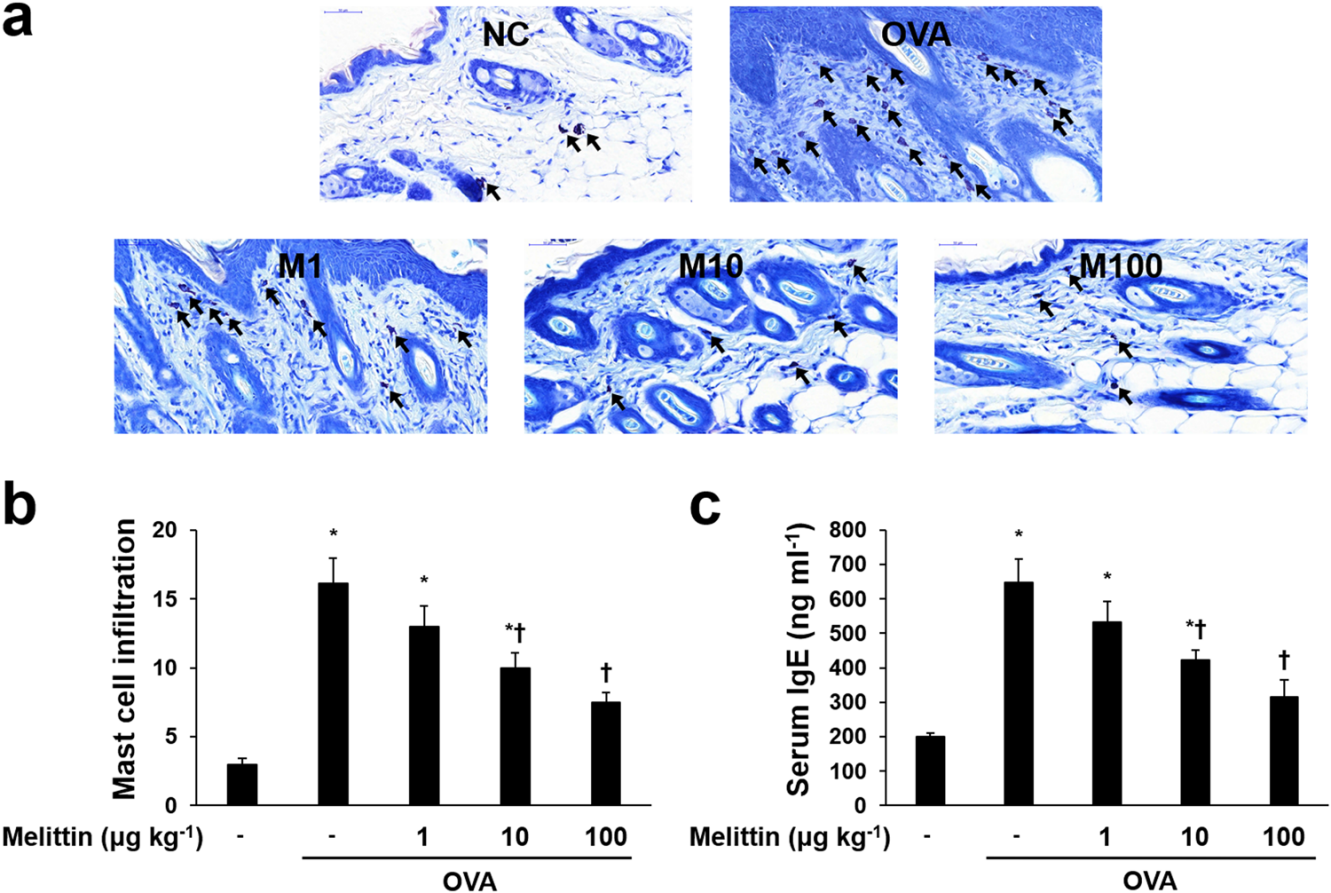
Effects of melittin on the OVA-induced increased mast cells and serum-IgE. (**a**) Representative images of histologic analysis with Giemsa staining showed that OVA increased the infiltrations of mast cells (arrows), whereas melittin decreased them. Scale bar = 50 μm. (**b**) The number of infiltrated mast cells was counted from at least five random fields per section at 400-fold magnification. The results are expressed as means ± SEM. (**c**) ELISA results demonstrate that melittin suppressed OVA-induced increased serum-IgE concentrations. The results are expressed as means ± SEM of three independent experiments. *p < 0.05 compared with the NC group. ^†^ *p* < 0.05 compared with the OVA group. NC: normal control; OVA: ovalbumin; M1, M10, and M100: 1, 10, and 100 μg kg^−1^ melittin; −: untreated.

To demonstrate the effects of melittin on OVA-induced increased IgE response, IgE enzyme-linked immunosorbent assay (ELISA) and counting the infiltrations of mast cells were performed. The infiltrations of mast cells were significantly increased in the OVA group compared with the NC group. However, compared with those in the OVA group, the infiltrations of mast cells in the melittin groups were remarkably reduced in a dose-dependent manner (Fig. 5a and b). Serum-IgE was measured with ELISA. The OVA increased serum-IgE, and it was decreased in a dose-dependent manner in the melittin treatment groups (Fig. 5c).

### Melittin decreases the OVA-induced expressions of inflammatory mediators in mouse dorsal skin

**Figure 6:**
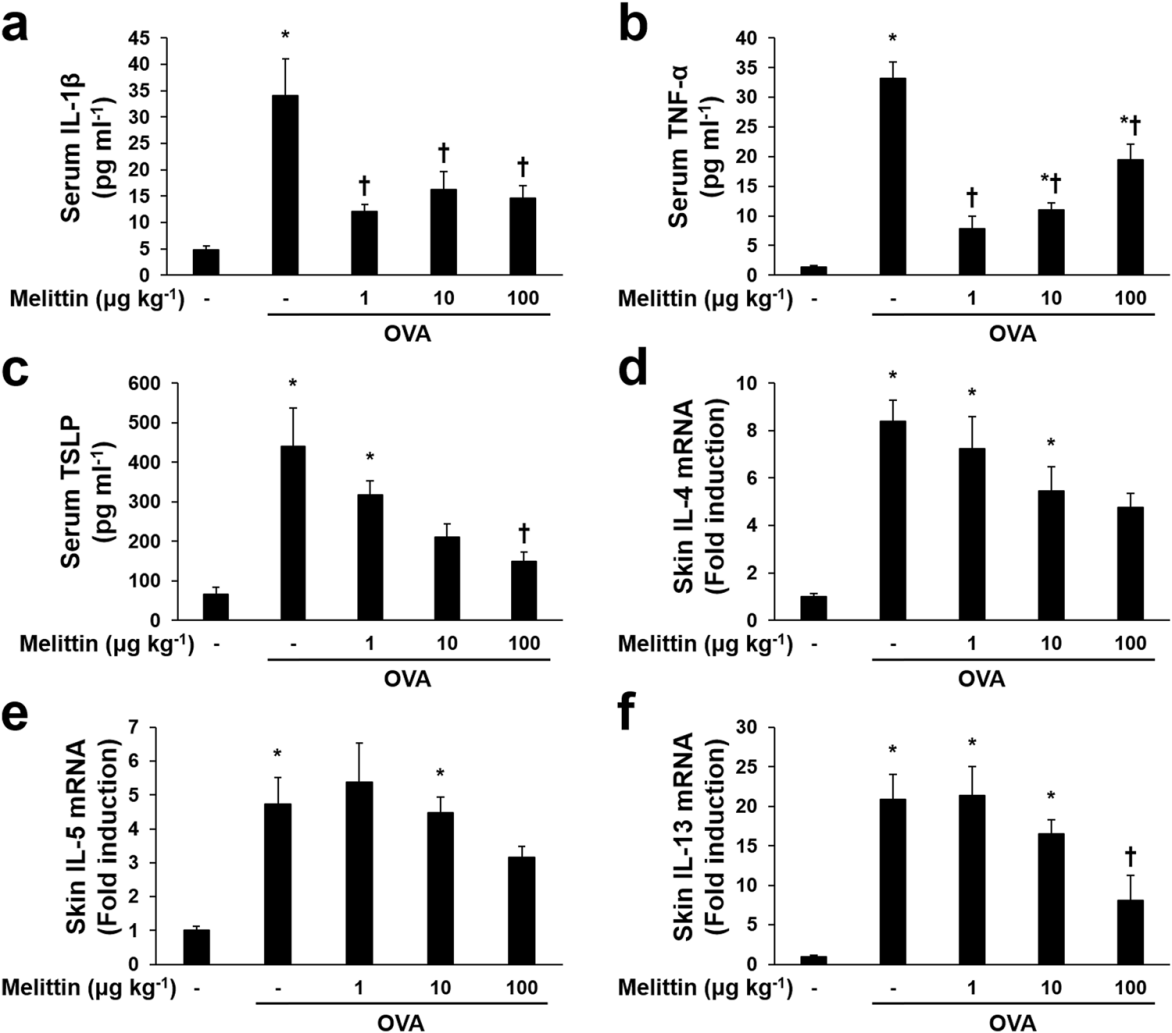
Effects of melittin on the OVA-induced inflammatory mediators. ELISA results show that the OVA-induced serum IL-1β (**a**), serum TNF-α (**b**), and serum TSLP (**c**) were decreased in the groups that received a suitable concentration of melittin. (**d,f**) Results of qRT-PCR demonstrate the effects of melittin on Th2 cytokines. The graphs summarize the analysis of the relative IL-4, IL-5, and IL-13 mRNA expressions, each normalized to GAPDH. All the results are expressed as means ± SEM of three independent experiments. *p < 0.05 compared with the NC group. ^†^ *p* < 0.05 compared with the OVA group; −: untreated.

Quantitative real-time polymerase chain reaction (qRT-PCR) and ELISA were performed to determine the effects of melittin on OVA-induced Th2 polarization and inflammation. Pro-inflammatory cytokines, such as IL−1β and tumor necrosis factor (TNF)-α, T cell-attracting chemokine TSLP, and Th2 cell-induced cytokines, such as IL-4, IL-5, and IL 13, were increased in the OVA group compared with the NC group (Fig. 6). The effects of melittin were slightly different in each case. In the case of IL-1β, all doses of melittin decreased the concentration of serum-IL-1β with the OVA group (Fig. 6a). The case of TNF-α was somewhat similar to that of IL-1β. The expression of serum-TNF-α was significantly suppressed in all melittin groups compared with the OVA group, while all melittin groups showed an increase in TNF-α in a dose-dependent manner, compared with the NC group (Fig. 6b). Serum TSLP was decreased in a dose-dependent manner in the melittin groups compared with the OVA group, but this difference was not significant, except for the 100 μg kg^−1^ melittin group (Fig. 6c). IL-4 mRNA expression in the skin lesion seemed to be reduced in melittin groups compared with the OVA group. However, there was not significant difference (Fig. 6d). Figure 6e shows that compared with the OVA group, all the melittin groups seemed to suppress IL-5 mRNA, except the 1 μg kg^−1^ of melittin group, but no significant difference was observed. The expressions of IL-13 mRNA were more noticeably reduced in the 100 μg kg^−1^ melittin group than in the OVA group (Fig. 6f).

### Effects of melittin on cell viability

**Figure 7:**
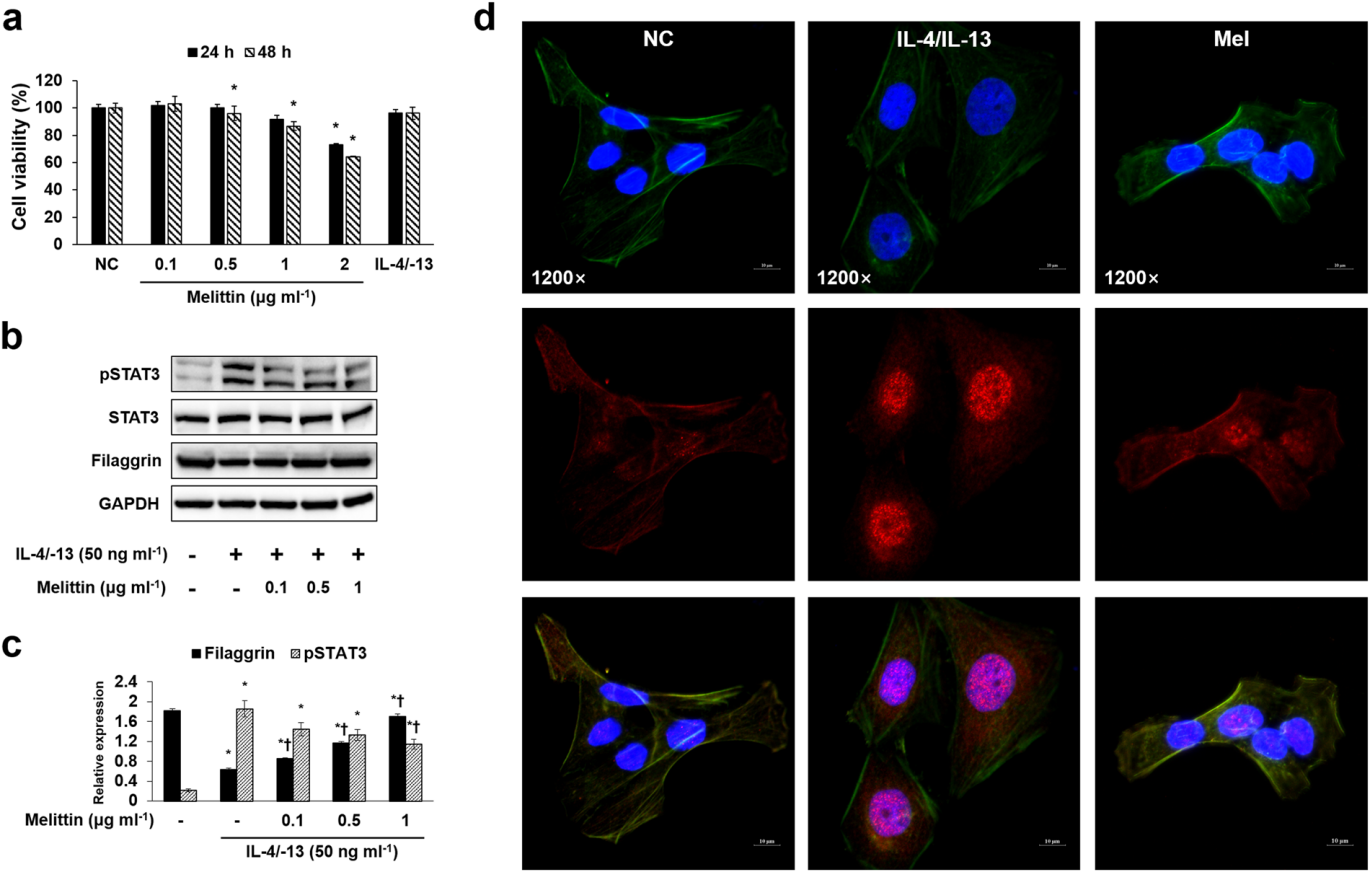
Melittin inhibits IL-4/IL-13-induced pSTAT3 activation and filaggrin deficiency in HaCaT cells. (**a**) CCK-8 assay results show the cytotoxic effect of melittin on HaCaT cells. The HaCaT cells were treated with 0.1, 0.5, 1, and 2 μg ml^−1^ of melittin, or 50 ng ml^−1^ each of IL-4 and IL-13 for 24 and 48 h. Results are expressed as means ± SEM of three independent determinations. *p < 0.05 compared with the NC group. (**b**) Representative cropped Western blot images show that melittin inhibited the IL-4/IL-13-induced protein expressions of pSTAT3, and it also improved IL-4/IL-13-induced filaggrin deficiency in the HaCaT cells. GAPDH was presented as loading control. The samples were derived from the same experiment and that blots were processed in parallel. +: treated; −: untreated. Full-length the blots can be found in Supplementary Fig. S1b. **(c)** The bar graph shows the quantitative signal intensity of filaggrin and pSTAT3 after normalization with GAPDH and STAT3, respectively. (**d**) Representative immunofluorescence images show the effect of melittin on the IL-4/IL-13-induced activation of pSTAT3 (labeled with Alexa Fluor 555, red). β-actin was labeled with Alexa Fluor 488 (green). The nuclei were labeled with Hoechst 33342 (blue). All the scale bars in the images are 10 μm. NC: normal control; IL-4/IL-13: 50 ng ml^−1^ each of IL-4 and IL-13; Mel: 1 μg ml^−1^ of melittin; 1200×: 1,200-fold magnification.

Cell counting kit (CCK)-8 assay was conducted to determine the cytotoxicity of melittin at different doses. The human keratinocytes (HaCaT) were treated with 0.1, 0.5, 1, and 2 μg ml^−1^ of melittin for 24 or 48 h. In the 24 h melittin treatment, HaCaT cell viability was reduced at 2 μg ml^−1^ of melittin. However, 0.1, 0.5, and 1 μg ml^−1^ of melittin did not alter HaCaT cell viability (Fig. 7a). In the 48 h BV treatment, HaCaT cell viability was reduced at 0.5, 1, and 2 μg ml^−1^ of melittin. In accordance with this result, the melittin treatment for the HaCaT cells was performed for 24 h, and 2 μg ml^−1^ of melittin was excluded in subsequent experiments.

### Melittin prevents filaggrin deficiency and STAT3 activation in human keratinocytes

Western blot results showed that IL-4 and IL-13 decreased filaggrin expression in HaCaT human keratinocytes, and they also increased signal transducer and activator of transcription (STAT)3 activation. Filaggrin expression was increased in a dose-dependent manner in the melittin treatment groups compared with the IL-4/IL-13 group. IL-4/IL-13-induced phosphorylated (p)STAT3 expression was decreased in the melittin treatment groups (Fig. 7b), and the same result was also found for immunofluorescence. The IL-4/IL-13-induced expression of pSTAT3 was inhibited by 1 μg ml^−1^ of melittin (Fig. 7c).

## Discussion

AD is a chronic skin disease characterized by pruritus and eczematous skin lesions, accompanied by immune responses dominated with Th2 cells in the acute phase, defects in the epidermal barrier, a thickened epidermis, and IgE-mediated sensitization to several antigens^1^. The mechanism involved in AD has not been fully understood. Currently, two main abnormalities are conjectured to underlie AD development, namely, epidermal barrier and immune defects, and these two are closely associated^42^. Therefore, therapies that target AD should consider not only epidermal barrier dysfunction but also immune responses.

Melittin, a major component of bee venom, has been known for its therapeutic effects in various diseases^43,44^. However, although the anti-inflammatory effects of melittin and bee venom on skin disease have been examined^40^, the beneficial effects of melittin on AD have yet to be studied. These effects of melittin on AD were therefore demonstrated in this study.

OVA is a major egg white protein. OVA-sensitized mice developed AD symptoms characterized by increased scratching behavior, skin thickening, infiltration of inflammatory cells, and upregulated expressions of Th2 cytokines, such as IL-4, IL-5, and IL-13^29^. In this study, OVA caused skin thickening, inflammatory infiltrate, and upregulated expressions of Th2 cytokines (IL-4, IL-5, and IL-13). Therefore, OVA is a useful allergen for developing an AD mouse model, and an OVA-sensitized mouse model may contribute to AD studies, such as the present one.

Filaggrin plays an important role in epidermal barrier function. Previous studies showed that the expression of filaggrin mRNA in keratinocytes was remarkably decreased in treatments of IL-4 and IL-13 *in vitro* ^45,46^, suggesting that Th2 cytokines contribute to the defect of epidermal differentiation^47^. In addition, the epithelial cell-derived cytokine TSLP, which contributes T cell populations that mature through the activation of antigen presenting cells, was also known to downregulate filaggrin expression^48^. These cytokines (IL-4, IL-13, and TSLP) can be upregulated by OVA stimulation^49,50^. Therefore, OVA is considered to have the ability to induce filaggrin deficiency through an increase in IL-4, IL-13, and TSLP expressions. In this study, melittin treatment ameliorated OVA-induced IL-4, IL-13, TSLP, and filaggrin deficiency. Th2 cytokines, such as IL-4 and IL-13, are also known as activators for STAT3, and the activation of STAT3 can cause the downregulation of filaggrin^51^. Therefore, confirming whether melittin inhibits STAT3 activation in keratinocytes is important to evaluate the cellular and molecular mechanism of melittin in filaggrin deficiency. In the study, melittin prevented IL-4/IL-13-induced filaggrin deficiency and STAT3 activation in keratinocytes. These results suggest that melittin may beneficially affect the epidermal barrier function by preventing filaggrin deficiency through a decrease in IL-4, IL-13, TSLP, and pSTAT3 expressions.

Another important feature in AD is the thickened skin resulting from the infiltration of activated T cells, eosinophils, mast cells, and monocytes/macrophages^3^. OVA-sensitized mice lesions are characterized by a thickened epidermis and dermis, as well as the infiltration of eosinophils, mast cells, macrophages, and CD4+ T cells^29,52,53^. In this study, histological analysis with H&E and Giemsa staining and immunohistochemical analysis with CD4 and CD11b showed that OVA induced epidermal and dermal thickening with increased inflammatory infiltrate. Furthermore, the OVA-induced thickened skin and increased inflammatory infiltrate were decreased in the melittin groups compared with the OVA group. These results suggest that melittin may ameliorate skin thickening through the regulation of the inflammatory infiltrate in AD.

Moreover, histological analysis with Giemsa staining showed that OVA caused the infiltration of mast cells in mice skin lesions. The OVA-induced infiltration of mast cells was inhibited in melittin-treated mice. Degranulation of the infiltrated mast cells plays a crucial role in IgE-mediated sensitization, releasing granules that lead to allergenic responses^27^. Exaggerated IgE-mediated sensitization is one of the main characteristics of AD^3^. Another feature of the OVA-sensitized mouse is their systemically elevated serum IgE level^54^. The ELISA result in this study showed that the serum-IgE concentration was increased by the OVA sensitization, and it was downregulated in the melittin treatment groups. These findings, including the inhibitory effects of melittin on mast cell infiltration and serum-IgE level, were suggestive that melittin can reduce exaggerated IgE response.

OVA activates the inflammatory cells, such as mast cells, dendritic cells, and Langerhans cells through IgE, and they contribute to allergic responses^26,52^. The activated mast cells release IL-1β, IL-4, IL-5, TNF-α, and several inflammatory mediators in the inflammatory site^55^. IL-4 and TNF-α increase vascular cell adhesion molecule-1 expression in endothelial cells, leading to the adhesion of several inflammatory cells to the inflammatory sites^56,57^. IL-1β, a pro-inflammatory cytokine, contributes a variety of immune reactions to promote mast cell activation and the production of Th2 cytokines^58,59^. IL-1β can also activate Th2 cells and enhance antibody production^60^. Thus, OVA stimulation can induce inflammatory cytokines, such as IL-1β, IL-4, IL-5, and TNF-α. In this study, OVA stimulation induced the following cytokines: IL-1β, IL-4, IL-5, and TNF-α. These results support the findings of previous studies^55,61^. Furthermore, IL-1β, IL-4, and TNF-α, except for IL-5, were significantly inhibited in the 100 μg kg^−1^ of melittin group compared with the OVA group. These results suggest that a suitable concentration of melittin may reduce inflammatory responses through the suppression of IL-1β, IL-4, and TNF-α in AD.

In conclusion, the OVA can contribute crucially to the AD-like cutaneous symptoms, such as thickened epidermis and dermis, inflammatory infiltration, filaggrin deficiency, exaggerated serum-IgE, and increased inflammatory mediators. Melittin can ameliorate these OVA-induced AD-like symptoms and allergic responses through suppression of inflammatory infiltration. Filaggrin deficiency is also improved through inactivation of pSTAT3 by melittin. Thus, this study can provide the molecular mechanism of melittin to improve the OVA-induced AD-like cutaneous symptoms and inflammatory mediators leading to the progression of AD. Furthermore, this study may be the first evidence that suitable concentration of melittin can use for anti-inflammatory effects on OVA-induced AD.

## Methods

### Cell culture and treatment

HaCaT (CLS, Eppelheim, Germany) cells were cultured in Dulbecco’s modified Eagle’s medium (DMEM) supplemented with 10% fetal bovine serum and 1% antibiotics at 37 °C in a humidified 5% CO2 incubator. The HaCaT cells were seeded at 1.0 × 10^6^ cells per 3 ml complete medium in a 100 mm TC-treated cell culture dish. The medium was changed 24 h later with a serum-free medium containing the indicated concentrations of melittin (0.1, 0.5, and 1 μg ml^−1^; Enzo Life Sciences, Farmingdale, NY, USA). After 1 h, the cells were co-treated with 50 ng/mL each of human recombinant IL-4 and IL-13 (R&D systems, Minneapolis, MN, USA). After 23 h, the cells were collected for the next experiments.

### Cell viability test

The cell viability of HaCaT was determined with CCK-8 assay (Dojindo, Kumamoto, Japan). The cells were seeded in a 96-well plate at 5.0 × 10^3^ cells per well and pre-incubated for 24 h. After pre-incubation, the cells were treated with melittin (0.1, 0.5, 1, and 2 μg ml^−1^) and 50 ng ml^−1^ each of IL-4 and IL-13 for 24 or 48 h. After treatment, 10 μL of WST-8 solution [2-(2-methoxy-4-nitrophenyl)-3-(4-nitrophenyl)-5-(2,4-disulfophenyl)-2H-tetrazolium, monosodium salt] was added to each well, and the cells were incubated for an additional 4 h at 37 °C. The cell viability values were measured by absorbance at 450 nm using a microplate reader.

### Animals

Six-week-old female BALB/c mice were used in the experiments. These mice were purchased from Samtako (Osan, Korea). Animal care and all experimental procedures were approved and conducted in accordance with the guidelines of the Institutional Animal Care and Use Committee of the Catholic University of Daegu (Approval number: DCIAFCR-160428-1-Y). After one week of acclimation, the 25 mice were used and randomly divided into five groups (five mice per group) as follows: untreated group (normal control, NC), OVA-sensitized group, and OVA-sensitized and melittin-treated group (1, 10, and 100 μg of melittin per kg of mouse bodyweight).

### Induction of atopic dermatitis

Figure 1a shows that AD was induced with some modifications, as previously described^29^. The mice in the group without NC were intraperitoneally inoculated with 10 μg of chicken OVA (grade V; Sigma, MO, USA) mixed with 4 mg of aluminum hydroxide (ImjectAlum; Thermo Fisher Scientific, MA, USA) in a volume of 200 μl three times at one-week intervals (i.e., on days 0, 7, and 14). The mice were anesthetized by isoflurane inhalation (Ifran; HANA Pharm, Seoul, Korea) by using RC2 Rodent Circuit Controller (VETEQUIP, CA, USA). During anesthesia, dorsal skin was shaved with an electric clipper and hair removal creams. After shaving, the mice were epicutaneously sensitized with OVA patches on day 14. The OVA patches were prepared with 1 cm^2^ of sterile gauze moistened with 100 μg OVA in phosphate-buffered saline (PBS). The OVA patches were attached on the shaved dorsal skin with a transparent dressing (Tegaderm; 3 M, MN, USA) for seven days (i.e., from days 14 to 20) and were changed daily. Then, the indicated concentration of melittin (1, 10, and 100 μg per kg mouse bodyweight) was injected intraperitoneally. The injection was performed twice a week. After melittin treatment, the OVA patches were attached again for a week. After the OVA treatment with patches, blood was collected by cardiac puncture from the mice, and the mice were sacrificed by CO_2_ asphyxiation. After sacrifice, their dorsal skins were excised for the next experiments. The dorsal skin sections were divided into three parts on the basis of use. One part was transferred into 10% formalin for histologic analysis. The other parts were transferred into liquid nitrogen as soon as possible and stored in a deep freezer at −70 °C before mRNA and protein extraction.

### Histological analysis

The dorsal skin specimens were fixed in 10% formalin for 24 h at room temperature. The fixed skin specimens were dissected, dehydrated, and embedded in paraffin. Multiple sections (4 μm thick) were mounted on slides. The sections on slides were stained with H&E to analyze the thicknesses of the epidermis and dermis and the inflammatory infiltrate; they were also stained with Giemsa to count mast cells. All slides were examined under a Pannoramic MIDI slide scanner (3DHISTECH, BU, Hungary). The thicknesses of the epidermis and dermis were measured from at least 10 random fields per section at 200-fold magnification with CaseViewer 1.4 software (3DHISTECH, BU, Hungary). The number of infiltrated mast cells was counted from at least five random fields per section at 400-fold magnification with CaseViewer 1.4 software.

### Immunohistochemical analysis

The paraffin-embedded tissue sections on slides were deparaffinized. The sections were then incubated with a primary antibody (1:100 dilution) for 1 h at 37 °C. The primary antibodies were anti-CD4 and anti-CD11b (Abcam, Cambridgeshire, UK). The signal was visualized using an Envision System (DAKO, CA, USA) for 30 min at 37 °C; 3,3′-diaminobenzidine tetrahydrochloride was used as the coloring reagent, and hematoxylin was used as the counter-stain. The slides were examined with a Pannoramic MIDI slide scanner, and the integrated optical density was analyzed with iSolution DT software.

### Enzyme-linked immunosorbent assay

The collected bloods by cardiac puncture were allowed to clot for 1 h at room temperature. The clots were removed by centrifugation (4,000 rpm, 20 min). Sera were obtained from the supernatants after centrifugation for ELISA. The concentrations of IgE, IL-1β, TNF-α, and TSLP in the sera were determined with an ELISA kit, according to the manufacturer’s instructions. The ELISA kits were purchased from Bethyl Laboratories (IgE; Montgomery, TX, USA) and R&D Systems (IL-1β, TNF-α, and TSLP).

### Quantitative real-time polymerase chain reaction

Total mRNA was extracted from the dorsal skin by using TRIzol reagent (Thermo Fisher Scientific, Waltham, MA, USA), according to the manufacturer’s recommendations. Reverse transcription reaction was performed with AccuPower RT Premix and Oligo dT18 (Bioneer, Daejeon, Korea), according to the manufacturer’s instructions. qRT-PCR was performed using LightCycler® nano instrument (Roche Applied Science, Mannheim, Germany) with TaqMan® Gene Expression Master Mix (Thermo Fisher Scientific). The qRT-PCR mixes contained 100 ng of cDNA and 1 μl each of FAM™ dye-labeled Taqman® Gene Expression Assay and VIC® dye-labeled Taqman® Endogenous Control (predesigned primers and probes; Thermo Fisher Scientific). The expression values of the target gene and the endogenous gene were simultaneously measured by detecting each of the FAM® dye and the VIC® dye. The measured expression values of each target gene were normalized to the expression values of each endogenous gene by using glyceraldehyde 3-phosphate dehydrogenase (GAPDH). The analyzed target genes are IL-4, IL-5, and IL-13.

### Western blot

Protein samples were prepared from the dorsal skin and the cultured HaCaT cells with a protein extraction buffer (Cell Lytic™ M; Sigma), according to the instruction manual. The protein concentration of the samples was measured with Bradford assay (Bio-Rad Laboratories, CA, USA), with the optimal density at 595 nm, by using a spectrophotometer. The protein samples were separated on precast gradient polyacrylamide gels (Bolt™ 4–12% Bis-Tris Plus Gels; Thermo Fisher Scientific) and transferred to nitrocellulose membranes (GE Healthcare) by using Bolt™ Mini Blot Module and Mini Gel Tank (Thermo Fisher Scientific), according to the manufacturer’s recommendations. The membrane was blocked in 5% bovine serum albumin. The blocked membrane was probed with a primary antibody and horseradish peroxidase-conjugated secondary antibody. Following a repeat of the wash step, the membrane was kept in enhanced chemiluminescence detection reagents (Thermo Fisher Scientific) for 1 min. Signal intensity was measured with an image analyzer (ChemiDoc™ XRS+; Bio-Rad Laboratories). The protein expression values were normalized to GAPDH expression values. The primary antibodies used were anti-filaggrin (#ab24584; Abcam), anti-STAT3 (#9139; Cell Signaling Technology, Danvers, MA, USA), anti-pSTAT3 (#9145; Cell Signaling Technology), and anti-GAPDH (#sc-32233; Santa Cruz Biotechnology, TX, USA).

### Immunofluorescence analysis

The HaCaT cells were seeded at a density of 1.5 × 10^5^ cells per well in a two-chamber slide (Eppendorf, Hamburg, Germany). After 24 h, melittin and IL-4/-13 were treated. The treated cells were washed with PBS and fixed with 4% paraformaldehyde for 20 min at room temperature. The fixed cells were treated with 0.1% Triton X-100 in PBS for 2 min to permeabilize. Following permeabilization, the cells were blocked in PBS containing 5% bovine serum albumin at room temperature for 1 h. After blocking, the cells were incubated with a diluted primary antibody overnight at 4 °C, and a secondary antibody for 4 h at room temperature. The nuclei were stained with Hoechst 33342 solution for 20 min. The slides were mounted using a fluorescence mounting medium (Dako, Santa Clara, CA, USA). To analyze the paraffin-embedded dorsal skin sections on the slides, deparaffinization was performed. After deparaffinization and antigen retrieval, the entire process described above was repeated, starting from the blocking process. The specimens were examined and photographed using a confocal microscope system A1 (Nikon, Tokyo, Japan). The antibodies used for immunofluorescence were anti-β-actin (Alexa Fluor 488 conjugate, #8833; Cell Signaling Technology), anti-STAT3 (#9139, Cell Signaling Technology), anti-filaggrin (#ENZ-ABS181; Enzo Life Sciences), and anti-mouse IgG (Alexa Fluor 555 conjugate, Thermo Fisher Scientific).

### Statistical analysis

All data are presented as means ± standard error of the mean (SEM). Statistical significance was tested by one-way analysis of variance with Tukey’s multiple comparison test. Differences with *p* < 0.05 were considered significant.

## Electronic supplementary material

Supplementary figure
