# Supplementary material for: Beneficial effects of melittin on ovalbumin-induced atopic dermatitis in mouse
Source: Sci Rep. 2017 Dec 15;7:17679. doi: 10.1038/s41598-017-17873-2 (PMC5732199; doi:10.1038/s41598-017-17873-2)
Supplement: Supplementary file 1 — Supplementary figure [file 41598_2017_17873_MOESM1_ESM.pdf]

Beneficial effects of melittin on ovalbumin-induced atopic dermatitis in mouse

Woon-Hae Kim[1], Hyun-Jin An[1], Jung-Yeon Kim[1], Mi-Gyeong Gwon[1], Hyemin Gu[1], Minji Jeon[1], Woo Jung Sung[1], Sang Mi Han[2], Sok Cheon Pak[3], Min-Kyung Kim[4] and Kwan-Kyu Park[1,*]

[1]Department of Pathology, College of Medicine, Catholic University of Daegu, Daegu, Korea

[2]Department of Agricultural Biology, National Academy of Agricultural Science, Jeonju-si, Korea

[3]School of Biomedical Sciences, Charles Sturt University, Bathurst, Australia

[4]Department of Pathology, College of Medicine, Dongguk University, Gyeongju-si, Korea

*Correspondence

Professor Kwan-Kyu Park, Department of Pathology, College of Medicine, Catholic University of Daegu, 33 Duryugongwon-ro 17-gil, Nam-gu, Daegu 42472, Korea. E-mail: kkpark@cu.ac.kr Tel.: +82 53 650 4149 Mobile: +82 10 8833 3231

Supplementary information

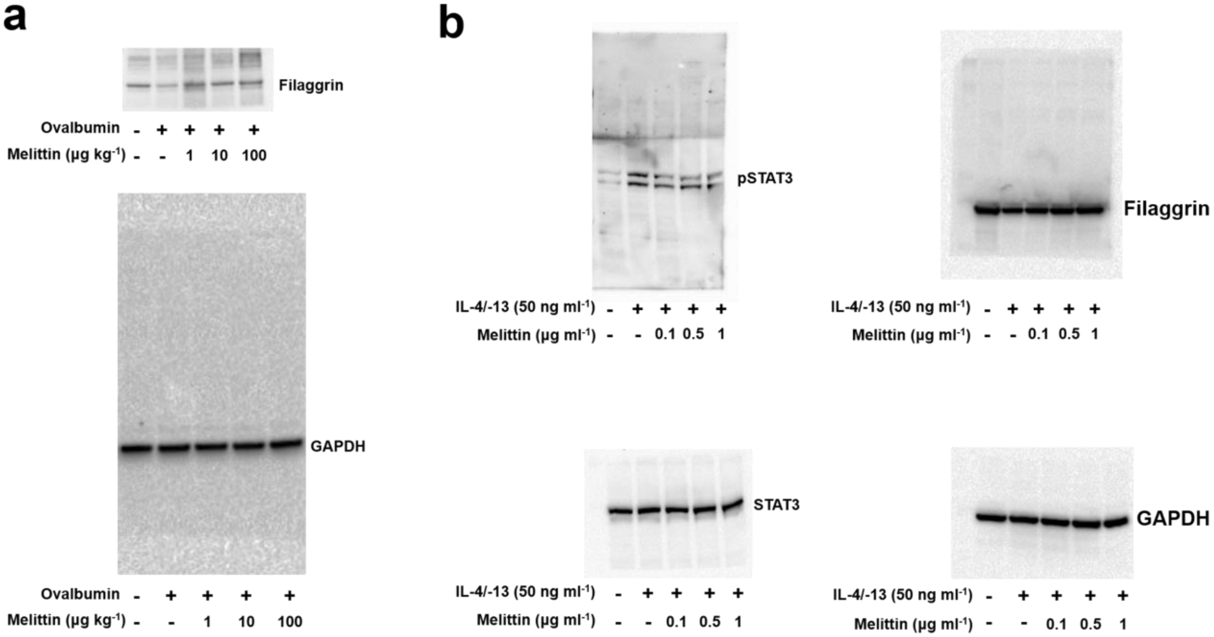

Supplementary figure S1. Full-length blots scanned by using ChemiDoc™ XRS+ image analyzer. Full-length blots of Figure 3 (**a**) and Figure 7 (**b**) are shown.
